# Use of Anecdotal Occurrence Data in Species Distribution Models: An Example Based on the White-Nosed Coati (*Nasua narica*) in the American Southwest

**DOI:** 10.3390/ani3020327

**Published:** 2013-04-17

**Authors:** Jennifer K. Frey, Jeremy C. Lewis, Rachel K. Guy, James N. Stuart

**Affiliations:** 1 Department of Fish, Wildlife and Conservation Ecology, New Mexico State University, Las Cruces, NM 88003, USA; 2 Department of Biology, New Mexico State University, Las Cruces, NM 88003, USA; E-Mail: jeremyclarklewis@gmail.com; 3 New Mexico Cooperative Wildlife Research Unit, Department of Fish, Wildlife and Conservation Ecology, New Mexico State University, Las Cruces, NM 88003, USA; E-Mail: guy@nmsu.edu; 4 New Mexico Department of Game and Fish, P.O. Box 25112, Santa Fe, NM 87504, USA; E-Mail: james.stuart@state.nm.us

**Keywords:** anecdotal data, climate, evidentiary standards, ecological niche models, Madrean, maximum entropy, occurrence records, species distribution models

## Abstract

**Simple Summary:**

We evaluated the influence of occurrence records with different reliability on predicted distribution of a unique, rare mammal in the American Southwest, the white-nosed coati (*Nasua narica*). We concluded that occurrence datasets that include anecdotal records can be used to infer species distributions, providing such data are used only for easily-identifiable species and based on robust modeling methods such as maximum entropy. Use of a reliability rating system is critical for using anecdotal data.

**Abstract:**

Species distributions are usually inferred from occurrence records. However, these records are prone to errors in spatial precision and reliability. Although influence of spatial errors has been fairly well studied, there is little information on impacts of poor reliability. Reliability of an occurrence record can be influenced by characteristics of the species, conditions during the observation, and observer’s knowledge. Some studies have advocated use of anecdotal data, while others have advocated more stringent evidentiary standards such as only accepting records verified by physical evidence, at least for rare or elusive species. Our goal was to evaluate the influence of occurrence records with different reliability on species distribution models (SDMs) of a unique mammal, the white-nosed coati (*Nasua narica*) in the American Southwest. We compared SDMs developed using maximum entropy analysis of combined bioclimatic and biophysical variables and based on seven subsets of occurrence records that varied in reliability and spatial precision. We found that the predicted distribution of the coati based on datasets that included anecdotal occurrence records were similar to those based on datasets that only included physical evidence. Coati distribution in the American Southwest was predicted to occur in southwestern New Mexico and southeastern Arizona and was defined primarily by evenness of climate and Madrean woodland and chaparral land-cover types. Coati distribution patterns in this region suggest a good model for understanding the biogeographic structure of range margins. We concluded that occurrence datasets that include anecdotal records can be used to infer species distributions, providing such data are used only for easily-identifiable species and based on robust modeling methods such as maximum entropy. Use of a reliability rating system is critical for using anecdotal data.

## 1. Introduction

Accurate information about the distribution of species is essential for their conservation and management [1]. Without this vital information, the species may not be considered when impacts of habitat alterations or other actions are considered for an area, or conservation and management efforts could be directed at areas where the species does not occur. Yet, in most cases species occurrence can only be sampled and hence distributions must be extrapolated. Traditionally, such extrapolation was accomplished via gestalt by a taxon expert, such as might be presented in a floral or faunal monograph [2]. However, the recent advent of niche-based species distribution modeling has provided rigorous quantitative means for extrapolating beyond known occurrence points [3,4]. These methods are attractive because they not only allow for habitat suitability of the organism to be predicted and mapped, but, they also allow for extrapolation of predicted occurrence beyond the study area and they provide information about the relationship between the species and the environment [5]. And yet, even these robust methods must be based on the fundamental underlying occurrence data, which are subject to a spectrum of errors.

Frey [2] provided a review of the nature of occurrence records, including types, problems with interpretation, and reasons for data gaps. Briefly, the most unambiguous type of occurrence record is based on preserved physical evidence, such as a museum voucher specimen, although even these can introduce error [2]. Other types of occurrence records are based on more equivocal forms of evidence, such as field observations of an animal or its sign. Because they cannot be independently verified, such records are considered anecdotal [6]. Occurrence records are subject to three dimensions of errors: the accuracy and precision of the spatial location, the spatial sampling of the records, and the reliability of the report. There has been a variety of recent studies that have evaluated the impact of spatial errors and bias on presence-only species distribution models (SDMs), which in general have found many methods to be robust, such as those based on maximum entropy [3,7,8,9,10,11].

In contrast to spatial errors, the impact of the reliability of occurrence records on the interpretation of species distributions has received little attention [1,2,6,12]. Although Lozier *et al.* [12] provided an extreme example of the problem of misidentification by comparing SDMs of the cryptozoid Sasquatch and the American black bear (*Ursus americanus*) and concluded that records of Sasquatch were a case of misidentification, no studies have evaluated influence of different severities in error of reliability on niche-based SDMs. Reliability refers to the degree to which an occurrence record can be trusted to be accurate. Thus, reliability primarily refers to the accuracy of the species’ identification [2,6], although it also may include potential for deceitfully manufactured information. Frey [2] presented a scheme of seven classes, ranging from verified to erroneous, for evaluating the reliability of species occurrence records (Table 1). In this scheme, most of the classes (*i.e.*, B-F) are anecdotal records. The scheme was based on three criteria that can influence accurate identification of a species, including observable diagnostic characteristics of the species, environmental conditions during the observation, and the observer’s knowledge about diagnostic characters of alternative potential species [2]. Consequently, an occurrence record would have a lower probability of reliability if it is of a cryptic species, observed during poor conditions (e.g., animal moving through shrubs at night), or made by a novice observer. Yet, while reliability reflects the inherent essence of an occurrence record, rarely has it been explicitly considered during distributional analyses [2,13,14]. Most commonly, all accumulated occurrence records are included in distribution analyses or modeling without regard to possible deficiencies in reliability. Thus, species distribution modeling typically proceeds by implicitly assuming that all records are reliable. This problem is likely heightened due to the increasing availability of large networked species occurrence data sets (e.g., Global Biodiversity Information Facility, NatureServe). 

McKelvey *et al.* [1] argued that the use of anecdotal data to infer species distributions for rare or elusive species can lead to substantial errors, which can have profound negative impacts on conservation. For example, they found that overestimating species ranges due to accepting erroneous records caused underestimation of extent of range losses resulting in a delay in conservation actions for the fisher (*Martes pennanti*) in the Pacific states, failure to recognize historical isolation and extirpation of the wolverine (*Gulo gulo*) in California resulting in an underestimation of loss of diversity and distribution in this species, and “resurrection” of the extinct ivory-billed woodpecker (*Campephilus principalis*) in the southeastern United States resulting in the expenditure of funds on costly conservation measures that otherwise could have been spent on species verified as extant. They argued that the proportion of false positive records will be higher for rarer species. Thus, they suggested that higher evidentiary standards based on verifiable data be used for determining the distribution and status of rare or elusive species. 

The overarching goal of this study was to explore how datasets of occurrence records that differ by inclusion of various classes of reliability influence the interpretation of the distribution of an elusive carnivore, the white-nosed coati (*Nasu narica*), in the American Southwest. The coati is morphologically and behaviorally (e.g., arboreal, diurnal, gregarious, vocal) distinctive and hence not likely to be misidentified by experienced biologists in the American Southwest [15]. The species reaches its northern range limits in southwestern New Mexico and southeastern Arizona [15]. However, due to apparent rarity or elusiveness, the status and distribution of coatis in the region has been a matter of long-running conjecture [16,17,18,19,20,21,22,23,24,25]. For example, in New Mexico it is currently known by only two specimens [26], while in Arizona numerous records in the central part of the state have been considered to represent wanderers [21,22].

**Table 1:** Classes for evaluating the reliability and precision of occurrence records for the white-nosed coati (*Nasua narica*) in Arizona and New Mexico. Reliability classes were adapted from Frey [2].

Class	Characteristics
**Reliability**	
A	Verified: An expert’s evaluation of preserved physical evidence, including photographs.
B	Highly Probable: An expert’s accurate observation, but no physical evidence is preserved.
C	Probable: A first-hand report of an observation that is likely to be accurate. Convincing details are provided.
D	Possible: A potentially inaccurate observation made by an expert due to poor conditions.
E	Questionable: First-hand report of a potentially inaccurate observation because of the observer’s lack of knowledge, suboptimal observation conditions, or the lack of supporting details, this class is not as convincing as class C.
F	Highly Questionable: Records that have a high potential of inaccuracy. Includes second-hand and unpublished reports.
G	Erroneous: Physical evidence verifies the reported species was misidentified.
**Precision**	
H	Actual location likely <30 m of coordinate
I	Actual location likely 30–500 m of coordinate
J	Actual location likely 500–1,000 m of coordinate
K	Actual location likely 1,000–2,000 m of coordinate
L	Actual location likely 2,000–3,000 m of coordinate
M	Actual location likely >3,000 m of coordinate

## 2. Methods

### 2.1. Occurrence Records

We compiled existing records of the coati from Arizona and New Mexico based on published literature, searches of 34 museum collections in the Mammal Networked Information System (manisnet.org/manis), records maintained by the Arizona Natural Heritage Program and New Mexico Department of Game and Fish, and information on observations of coatis from biologists and other people in Arizona and New Mexico. Redundant locality records were eliminated by preferentially eliminating records with lower precision or reliability. Occurrence records were georeferenced as a latitude and longitude coordinate (World Geodetic System of 1984) using Delorme Topo U.S.A. version 5.0 topographical maps based on our interpretation of locality information contained in the record. These coordinates were projected into the USA Contiguous Albers Equal Area Conic (USGS version) projection using the 1983 North American Datum and given a UTM coordinate for use in ESRI ArcGIS Desktop 9.3. Georeferenced coordinates were ranked by precision (Table 1), which represented the likely deviation of the coordinate relative to the actual location. Records were ranked by reliability according to the system described in Frey [2] (Table 1). In this system, reliability is the confidence with which a record can be accepted and is based on presence of diagnostic features in the species, the observer’s knowledge of diagnostic characteristics of the focal species in comparison with other species, and conditions (e.g., duration of observation, lighting, presence of visual obstructions) under which the observation occurred. For purposes of this study, we defined an expert as an individual who regularly worked with, and could accurately identify, different types of wildlife within the American Southwest. Further, we regarded the coati as a unique species with a suite of obvious diagnostic morphological and behavioral characters that could allow accurate identification by knowledgeable observers.

**Figure 1:**
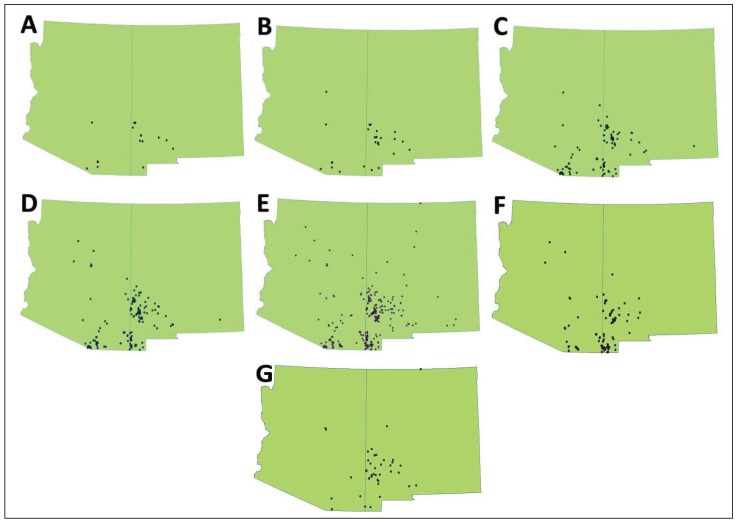
Comparison of subsets of occurrence records that differ in reliability and precision (see Table 3, Table 4) for the white-nosed coati (*Nasua narica*) in Arizona and New Mexico: (**A**) very conservative, (**B**) conservative, (**C**) best a priori, (**D**) moderate, (**E**) liberal, (**F**) poor precision, (**G**) poor reliability.

Occurrence records exhibit simultaneous errors in reliability and spatial precision (Table 2). Species distribution models based on maximum entropy methods have been shown to be robust to location errors up to at least 5 km (using 100 m spatial resolution of environmental layer), with the upper limit unknown [11]. Further, deletion of records with large spatial error reduces sample size, which can negatively impact the SDM [27]. Consequently, spatial errors are rarely acknowledged or controlled for in species distribution modeling even though it is recognized that some museum data may have large errors [11]. Regardless, the influence of spatial error on SDMs has not been extensively evaluated and we wanted to insure that our results accounted for this error. Because this study was based on real data, it was not possible to hold precision constant for all reliability classes. Consequently, we created seven different datasets of occurrence records, that varied in reliability and precision, upon which the SDMs were based (Figure 1, Table 3, Table 4). 

**Table 2:** Number of occurrence records of the white-nosed coati (*Nasua narica*) in Arizona and New Mexico in each pair-wise combination of the reliability and precision classes (see Table 1). Sample sizes in this table may not match the sample sizes used in the MaxEnt models (see Table 3, Table 4) because the software eliminates spatially redundant records.

	Reliability						
**Precision**	**A**	**B**	**C**	**D**	**E**	**F**	total
**H**	18	58	10	1	3	2	92
**I**	12	33	13	0	7	7	72
**J**	9	17	10	1	3	5	45
**K**	6	14	4	0	2	4	30
**L**	4	11	1	0	0	1	17
**M**	6	31	7	3	3	11	61
total	55	164	45	5	18	30	317

Five of these datasets were constructed to represent realistic scenarios about how occurrence records might be selected for inclusion in studies aimed at interpreting species distributions. The liberal dataset included all occurrence records regardless of reliability and precision. The best a priori dataset was based on deductive reasoning about what levels of error would be acceptable and included reliability based on verified records and observations by an expert (reliability A-B) as well as high precision (precision H-I). Although the best a priori dataset contained anecdotal data (*i.e.*, reliability class B), we regarded these records as having relatively low error because coatis are distinctive and readily identifiable by experts. The moderately restrictive dataset was more liberal than the best a prior dataset, but excluded the higher classes of error (reliability A-C; precision H-J). Two conservative datasets included only verified records (reliability A) with high precision (very conservative: precision H; conservative: precision H-I). In addition, to explicitly evaluate how error in reliability and spatial precision influenced the models, we developed a poor reliability and a poor precision dataset. These were identical to the best a priori dataset with exception that the poor reliability dataset included only the least reliable records (reliability C-F) while the poor precision dataset included only the least spatially precise records (precision J-M).

**Table 3:** Occurrence records, area-under-the-curve (AUC) statistics, and variable contributions to bioclimatic ecological niche models for the white-nosed coati (*Nasua narica*) in Arizona and New Mexico.

Models		Occurrence records ^1^			AUC			Variable contributions (%) ^2^											
No.	Name	Reliability	Precision	N	Mean Training	Mean Test	SD	Bio 3	Bio 4	Bio 6	Bio 8	Bio 9	Bio 10	Bio 13	Bio 14	Bio 15	Bio 16	Bio 17	Bio19
1	Very Conservative	A	H	18	0.945	0.933	0.025	39.0 **	5.8 *	19.5	1.8			22.7		1.2		5.1	4.9
2	Conservative	A	H-I	30	0.967	0.935	0.029	22.9 **	14.3 *		3.9	21.5			2.4	14.6	7.2	1.0	12.3
3	Best A Priori	A-B	H-I	103	0.967	0.956	0.013	32.3 *	13.7 **		2.3	19.3			0.9	14.8	6.4	0.4	10.0
4	Moderate	A-C	H-J	153	0.971	0.954	0.013	37.7 *	8.8 **		4.1	14.2			1.9	11.7	7.9	0.5	13.2
5	Liberal	A-F	H-M	279	0.958	0.934	0.014	38.5 *	12.1 **		4.5	13.2	2.6			12.2	5.5	1.2	10.2
6	Poor Reliability	C-F	H-I	42	0.950	0.909	0.038	63.2 *	2.4 **		0.5	15.0			3.3	7.3	4.7	0.3	3.2
7	Poor Precision	A-B	J-M	89	0.955	0.944	0.022	19.2 *	12 **	15.4	5.9					14.3	7.7	3.0	22.5

^1^ See Table 1 for definitions of reliability and precision classes. ^2^ Bio 3: isothermality, Bio 4: temperature seasonality, Bio 6: minimum temperature of coldest month, Bio 8: mean temperature of wettest quarter, Bio 9: mean temp of driest annual quarter, Bio 10: mean temperature of warmest quarter, Bio 13: precipitation of wettest month, Bio 14: precipitation of driest month, Bio 15: precipitation seasonality, Bio 16: precipitation of wettest quarter, Bio 17: precipitation of driest quarter, Bio 19: precipitation of coldest quarter. Asterisks indicate the most important (*) and second most important (**) variables indicated via the jackknife test.

**Table 4:** Occurrence records, area-under-the-curve (AUC) statistics, and variable contributions to biophysical ecological niche models for the white-nosed coati (*Nasua narica*) in Arizona and New Mexico.

Models		Occurrence records ^1^			AUC			Variable contributions (%) ^2^								
No.	Name	Reliability	Precision	N	Mean Training	Mean Test	SD	Land-cover	Distance To Springs	Distance To Streams		Distance To Lakes		Slope	Elevation	Road Density
8	Very Conservative	A	H	18	0.972	0.878	0.067	50.7 *	27.1 **	2.1	6.2		1.0		0.3	12.7
9	Conservative	A	H-I	30	0.969	0.920	0.041	2.9 *	26.2 *	16.3	12.4		9.6		5.7	26.9 **
10	Best A Priori	A-B	H-I	115	0.974	0.941	0.082	28.6 *	26.2 **	9.7	6.6		12.1		9.5	7.3
11	Moderate	A-C	H-J	168	0.965	0.934	0.018	26.7 *	28.2 **	12.1	6.7		9.4		8.4	8.3
12	Liberal	A-F	H-M	299	0.954	0.926	0.015	30.6 *	24.1 **	11.6	3.4		10.1		8.7	11.5
13	Poor Reliability	C-F	H-I	42	0.953	0.876	0.046	37.7 *	22.3 **	7.7	5.6		7.4		5.6	13.8
14	Poor Precision	A-B	J-M	91	0.965	0.937	0.022	51.4 *	13.4 **	10.9	1.5		7.0		3.3	12.5

^1^ See Table 1 for definitions of reliability and precision classes. ^2^ Asterisks indicate the most important (*) and second most important (**) variables indicated via the jackknife test.

### 2.2. Model Development

The study area was confined to Arizona and New Mexico and bounded by 30.480826° and 38.138614° latitude, and −102.546856° and −115.882382° longitude. Explanatory power and predictive performance of bioclimatic niche models can be improved by including land-cover variables [28]. Consequently, in order to produce the most biologically relevant distributional information, we created SDMs for the coati by combining separate ecological niche models (ENMs) based on bioclimatic and biophysical variables [29]. For the bioclimatic ENMs we generated 19 biologically relevant variables from the WorldClim version 1.3 dataset that represented annual trends, seasonality, and extreme conditions in current climate (*i.e.*, 1950–2000) and that are routinely used in ecological niche modeling [29,30]. These variables were at a 1-km spatial resolution and were derived from monthly temperature and precipitation values. Data layers for the biophysical ENMs were at 240 m resolution and included land-cover [31], elevation and slope [32], road density [33], and distance from streams, springs, and lakes [34]. Descriptions of land-cover can be found at http://earth.gis.usu.edu/swgap/legenddataquery.php.

To develop ENMs we used a maximum-entropy algorithm, MaxEnt version 3.3.1 [35,36], which was specifically developed for use with presence-only occurrence data and which has been shown to be robust and accurate for developing models for rare organisms where occurrence records are few in number or spatially biased [8,9,10,11,37]. Our methods generally followed Calkins *et al.* [29] with exception that we used a lower threshold for identifying correlated climate variables (*r* ≥ |0.70|). We used the MaxEnt jackknife test of variable importance to determine which variables had the most useful information.

We combined the bioclimatic ENM and the biophysical ENM for each subset of occurrence records to produce a SDM, which was the area of overlap between the bioclimatic and biophysical ENMs. We used the equal test sensitivity and specificity threshold (*i.e.*, sensitivity and 1-specificity are equal) to converted the logistic probability values of each pixel in the bioclimatic ENMs into a binary map of suitable or unsuitable climate. This threshold was chosen in order to balance the errors of omission and commission in the model. For visual interpretation of the spatial model, we did not convert the biophysical ENMs to a binary format in order to preserve maximum information content.

### 2.3. Model Evaluations and Comparisons

We evaluated the performance of each model with area-under-the-curve (AUC) values, which is a measure of the threshold-independent relationship between the proportions of pixels correctly and incorrectly classified [35,36,38]. Models with AUC values closer to 1.0 (*i.e.*, perfect prediction) are better at distinguishing between presences correctly classified and non-presences incorrectly classified [28]. We focused on the AUC value of the test data (AUC_test_) because it is the most commonly used metric of model quality, it generally does not suffer from problems of overfitting as does AUC_train_, and it exhibited no significant differences (based on overlap of error bars) in a variety of model performance criteria compared to other approaches (e.g., AIC_c_) [39]. 

We compared models based on different subsets of occurrence records using AUC_test_. We used a modified critical ratio test [40] to compare the most different AUC values. For the bioclimatic ENMs we used the initial global models based on all 19 variables to insure that tests were based on the same set of variables. We calculated Kendall rank correlation coefficients in R version 2.12.0 [41] and related them to a table in Hanley and McNeil [42] to derive an adjusted correlation coefficient (*r*). We incorporated *r* into the critical ratio test following Pearce and Ferrier [40]. We performed z-tests using the resulting z-scores and using an α = 0.05. 

We compared differences in the spatial models through visual assessments and by evaluating percent similarity between pairs of models. Percent similarity was calculated by determining the proportion of 150,000 random points across the study area that intercepted pairs of SDMs being compared. For this analysis, the SDMs were created based on binary biophysical ENMs produced in the same manner as the binary bioclimatic ENMs using the equal test sensitivity and specificity threshold. 

## 3. Results

We obtained 317 unique occurrence records for *N. narica* (Table 2, Figure 1). Only one dataset (very conservative) had a small sample size (*i.e.*, <30 records), which can cause potential inaccuracies in SDMs [10,37]. All other models had adequate or large sample sizes (*i.e.*, >100 records) [10]. Further, 94.3% of records were from 1950 or later (79.2% since 1990) and hence the vast majority were within the time frame of the climate and land-cover data. All bioclimatic and biophysical ENMs had high (*i.e.*, >0.90) AUC_test_ values except the very conservative and poor reliability biophysical models (models 8 and 13; Table 3, Table 4). For both the bioclimatic and biophysical ENMs the best performing models were those that included a low to moderate classes of error in reliability and precision, with the best a priori model performing best. Model performance declined with either decreasing sample size (the very conservative and conservative models) or increasing error (liberal model). While the poor precision model performed almost as well as the best a priori model, the poor reliability model was the worst performing model. 

For the bioclimatic ENMs, isothermality (*i.e.*, Bio 3) had the highest variable contribution in all models, except model 7 (*i.e.*, poor precision model; Table 3). Other variables with significant contributions (*i.e.*, >5%) in most models (*i.e.*, models 2–5) included: mean temperature of the driest annual quarter (Bio 9), precipitation and temperature seasonality (Bio 15 and 4, respectively), and precipitation of the coldest and wettest quarters (Bio 19 and 16, respectively). The poor precision model (model 7) was similar in variable contributions to these models with exception that mean temperature of the wettest quarter (Bio 8) was also important. In contrast, the very conservative (model 1) and poor reliability (model 6) models markedly departed from these patterns in idiosyncratic ways. Regardless of the datasets used, the jackknife tests indicated that isothermality (Bio 3) and temperature seasonality (Bio 4) were always the first or second most important variables. 

For the biophysical ENMs, with a few exceptions, all variables were significant (>5%) contributors to all models (Table 4). The highest variable contributions in all but one of the biophysical models were land-cover and distance to springs, which together ranged from 55–78% of contribution to those models. The exception was the conservative model (model 9), wherein road density had the highest variable contribution and land-cover had the lowest contribution. In other models, road density was <14% contribution. The jackknife tests indicated that land-cover type, followed by distance to springs, were the most important variables (except for in the conservative model where road density was the second most important variable). Land-cover types with the highest suitability for coatis included Madrean encinal, Madrean pinyon-juniper woodland, and Mogollon chaparral, despite these accounting for only 21.4% of the area of suitable habitat (Table 5).

**Table 5:** Mean suitability of important land-cover types for the white-nosed coati (*Nasua narica*) in Arizona and New Mexico based on the best *a priori* set of occurrence records (see Table 3, Table 4). Only land-cover types accounting for >1% area of suitable habitat are included.

Land-cover type	Proportion of area of suitable habitat (%) ^1^	Mean habitat suitability (%)
Madrean Encinal	6.3	47.1
Madrean Pinyon-Juniper Woodland	12.6	40.7
Mogollon Chaparral	2.5	38.8
Chihuahuan Mixed Salt Desert Scrub	2.5	34.7
Madrean Lower Montane Pine-Oak Forest and Woodland	1.4	34.6
Apacherian-Chihuahuan Mesquite Upland Scrub	10.6	22.6
Apacherian-Chihuahuan Semi-Desert Grassland and Steppe	28.2	18.9
Chihuahuan Creosote, Mixed Desert and Thorn Scrub	10.4	16.7
Southern Rocky Mountain Ponderosa Pine Woodland	8.5	14.6
Colorado Plateau Pinyon-Juniper Woodland	5.1	13.9
Chihuahuan Stabilized Coppice Dune and Sand Flat Scrub	1.2	8.3
Sonoran Paloverde-Mixed Cacti Desert Scrub	2.8	5.9

^1^ The proportion of suitable habitat was the total number of 1,000 m^2^ map pixels with a certain land-cover type divided by the total map pixels (59,123) indicated as having a non-zero suitability in the biophysical ecological niche model.

**Figure 2:**
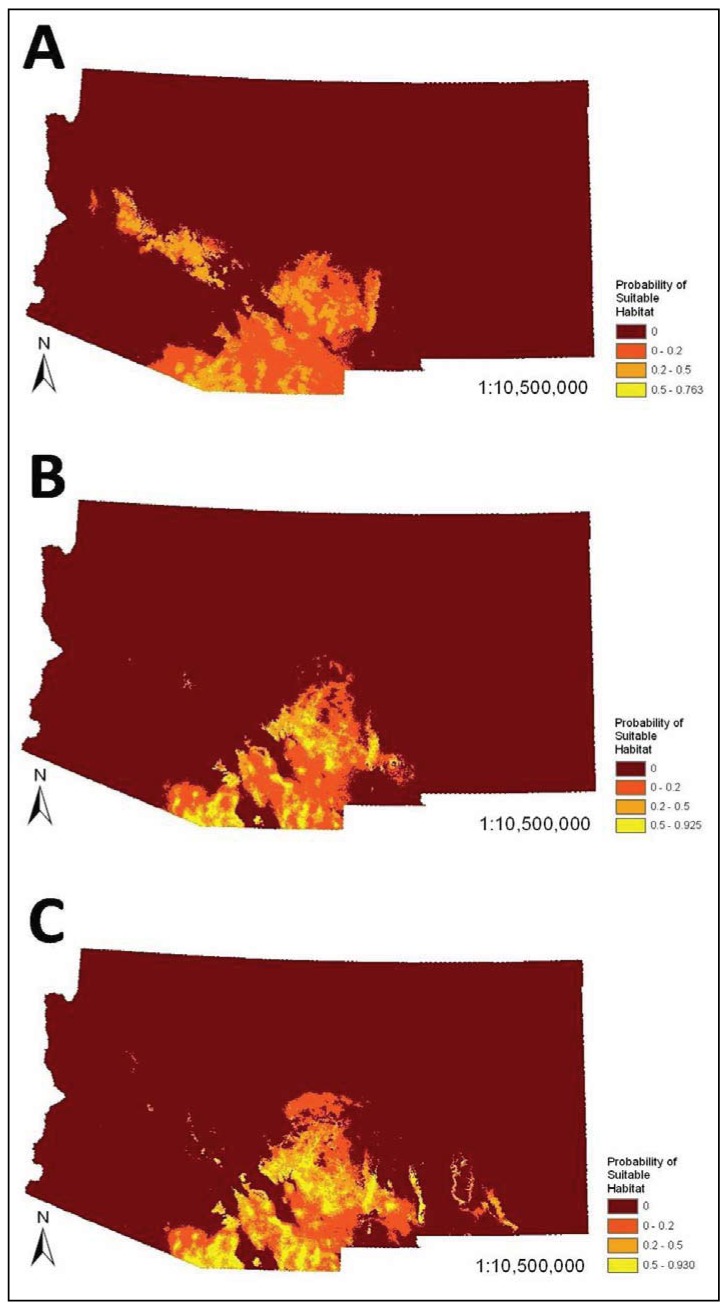
Species distribution models (*i.e.*, combined bioclimatic and biophysical ecological niche models (ENMs)) for the white-nosed coati (*Nasua narica*) in Arizona and New Mexico based on different subsets of occurrence records (see Table 3, Table 4): (**A**) very conservative, (**B**) best a priori, and (**C**) liberal.

The SDMs (*i.e.*, combined bioclimatic and biophysical ENMs) predicted occurrence of coatis primarily in southeastern Arizona and southwestern New Mexico (Figure 2). There was minor variation in the spatial models. For example, the model that visually departed from the others the most was the very conservative model, which predicted occurrence of coatis in the Arizona Central Highlands, which is a northwest trending mountainous region south of the Colorado Plateau in central Arizona. The liberal model slightly differed mainly in predicating mid elevations of mountain ranges and escarpments in southeastern New Mexico. However, percent similarity between the very conservative SDM and all other SDMs based on less reliable datasets were all very high (94% to 96.1%). In addition, the AUCs among all pair-wise comparisons of models within the bioclimatic or biophysical sets were not significantly different (*P* > 0.05), with exception of a comparison between the most dissimilar bioclimatic models (*i.e.*, very conservative model *versus* moderate model; *Z* = 2.678, *P* = 0.008).

## 4. Discussion

### 4.1. Influence of Reliability of Occurrence Records

With exception of the very conservative SDM, we found only minor variation in the predicted distribution of the coati based on datasets that varied in their inclusion of different classes of reliability. Reason for the similarity in predicted distribution among the models was likely because coatis are highly distinctive in appearance and behavior and are unlikely to be misidentified by careful or knowledgeable observers. In addition, because this is an unusual and relatively rare species in the United States, encounters with it are likely to be remembered, which probably contributed to the relatively large number of records reported and ranked as highly probable (Table 2). However, despite our findings that datasets that included anecdotal data had little influence on SDMs for the coati, we recommend that attempts to infer distribution of organisms make effort to evaluate reliability of the occurrence records and exclude those deemed too unreliable. For instance, distributions of species with fewer or more subtle diagnostic characters are more likely to be influenced by an accumulation of records based on misidentifications [1,12]. Indeed, we strongly suspect that even some of our occurrence records were based on misidentification (*i.e.*, they probably were of ringtail [*Bassariscus astutus*]). Further, it is expected that the proportion of false positives would be higher in extralimital locations. However, the maximum entropy procedure we used functions to assign low probabilities to such unusual occurrences and hence eliminates them from the final spatial models (e.g., compare Figure 1(e) with Figure 2(c)). Certainly, additional research is needed on the influence of reliability on species distribution modeling.

The very conservative dataset was included to illustrate the results if only the most reliable and precise occurrence records were included—that is, those based on physical evidence and location with <30 m error (*i.e.*, location recorded with GPS). The major difference of the SDM based on this dataset was that it predicted, though with low probability (<0.5), that the Arizona Central Highlands physiographic region was suitable for coatis, whereas other datasets did not. This difference is attributed to the small sample size (*N* = 18) of this dataset, which also is reflected by the lowered AUC_test_ for the biophysical variables (Table 4). Maxent is often considered the most robust method for generating SDMs based on small sample sizes, and it can generate useful models with as few as 5–10 records [8,9,10,37]. However, studies have shown that small samples also can cause Maxent to over-predict distribution and that such results are highly influenced by location of included records [8]. Thus, small sample size is a limitation that can challenge inferences about species distributions. Consequently, the judicious inclusion of occurrence records with greater degrees of error in reliability and spatial precision can function to increase sample sizes to improve knowledge about distribution. In so doing, model performance should be evaluated, but it also should be recognized that models that retain high quality in predicting geographic distribution may suffer from reduced ability to predict important environmental niche variables [39]. 

Areas of over-prediction in SDMs based on small sample sizes have led to the discovery of populations in new distributional areas [43,44]. These areas are interpreted as regions with environmental conditions similar to where the species occurs, and hence represent areas to target future surveys [8,9,44]. Climatically, the Arizona Central Highlands differs from southeastern Arizona in that it is cooler and wetter, with the majority of precipitation occurring during the winter rather than summer [45]. As a consequence, for example, Madrean encinal does not occur in the Central Highlands [31]. Thus, the Central Highlands may represent an area of marginal habitat for coatis that is occupied less frequently in space and time. However, the very conservative SDM suggests that additional inventory work is needed to confirm status of the coati in the Central Highlands.

### 4.2. Determinants of Coati Distribution

**Figure 3:**
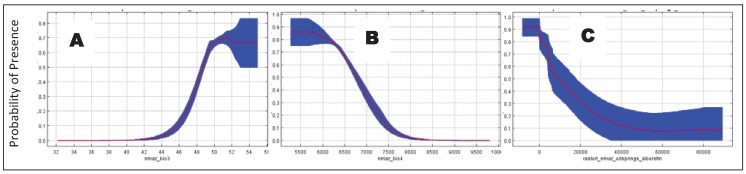
Response curve of the Maxent logistic probability of presence of the white-nosed coati (*Nasua narica*) in Arizona and New Mexico based on the best a priori subset of occurrence records with (**A**) isothermality (Bio 3), (**B**) temperature seasonality (Bio 4), and (**C**) distance to springs.

Evenness of temperature is a primary climatic determinant of coati distribution at its northern range limits in the American Southwest. Isothermality and temperature seasonality were the most important variables in all bioclimatic ENMs. Isothermality (*i.e.*, diurnal temperature range/temperature annual range × 100) is a measure of the magnitude of day-night temperature oscillation in relation to summer-winter temperature oscillation such that a value of 100 represents conditions where the daily temperature range is equal to the annual temperature range. In contrast, temperature seasonality (*i.e.*, standard deviation of monthly temperature) is a measure of the annual variation in temperature. Both of these variables exhibited strong, nearly threshold relationships with probability of coati occurrence (Figure 3(a,b)). High probability of coati occurrence was at locations with high isothermality and low temperature seasonality. 

**Figure 4:**
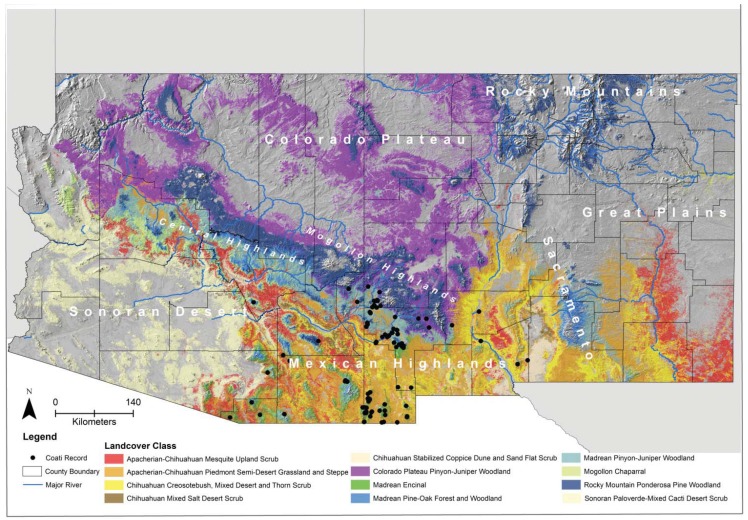
Map of land-cover types associated with the white-nosed coati (*Nasua narica*) in Arizona and New Mexico (see Table 5 for habitat suitability values for each land-cover). Dots represents observations of observations of coatis that included juveniles, pregnant females, or groups of three or more individuals, suggesting reproduction. Labels are physiographic regions.

Land-cover was the most important biophysical variable in determining coati distribution in the American Southwest. The land-cover types with the highest suitability for coatis form a natural transition at mid-elevations (*i.e.*, from highest to lowest elevation: Madrean pinyon-juniper woodland, Madrean encinal, Mogollon chaparral) between Madrean pine-oak forest at higher elevations and desert or arid grasslands at lower elevations (Figure 4). Indeed, the mean elevation of all verified (class A) and highly probable (class B) occurrence records was 1,611 m (range = 704–2,807; SD = 314.7; 95% confidence interval = 1,570–1,653; N = 219), which is similar to prior reports from Arizona [19,21]. Madrean woodland and chaparral communities are found in areas with mild winters, which directly reflects the bioclimatic determinants (*i.e*., evenness of temperature) of coati distribution [46]. Such areas are dominated by a diversity of evergreen shrub and tree species, such as mountain mahogany (*Cercocarpus* spp.), manzanita (*Arctostaphylos*), oaks (*Quercus*), pinyon pines (*Pinus*), and junipers (*Juniperus*), and primarily having centers of distribution in the Sierra Madre, Mexico. Thus, the coati would appropriately be considered a Madrean species that reaches its northern limit in the American Southwest. However, it should be noted that coatis are not limited to Madrean woodland and chaparral habitats; other land-cover types with high suitability included various types of desert scrub, semi-desert grassland, woodland, and pine forest (Table 5). These habitat relationships have been noted by previous researchers [21]. 

Distance to springs was the second most important biophysical variable determining distribution of coatis in the American Southwest. The response curve indicated that coati presence was more likely nearer to springs (Figure 3(c)). This is an arid region with relatively few perennial water sources. Consequently, springs likely represent a critical resource for coati occurrence. For instance, in Arizona home ranges of coatis tended to be associated with perennial water sources [47,48,49] while in a tropical dry forest in Mexico home range size was best explained by dispersion of water sources during the dry season [50]. Other studies have reported close association of coatis with springs and riparian areas in the American Southwest [19,22,48]. Our analyses also indicated riparian habitats were important for coatis. For instance, several riparian cover types had high mean habitat suitability (North American warm desert lower montane riparian woodland and shrubland, 38.0; Rocky Mountain lower montane-foothill riparian woodland and shrubland, 25.1; North American warm desert riparian mesquite bosque, 22.2; North American warm desert riparian woodland and shrubland, 18.8). Yet, together these made up <0.5% of the area of suitable habitat.

Response curves indicated an increasing probability of coati occurrence with increasing road density. This pattern is expected because roads provide access and opportunity for people to observe coatis. However, the relative importance of road density varied among the models (Table 4). For instance, road density was the most important variable in the conservative model, but it was the third most influential variable in the very conservative model, and one of the least influential variables in the best a priori model. One might postulate that this was due influence of specimens found dead on roads, but that is not the case. Of the 30 occurrence records in the conservative dataset, only 23% were specimens (the remainder were photographs) and only one appeared to have been taken from a road. Further, of the 12 occurrence records added to the conservative model in class I, only one was from near an urban area and only three appeared to be taken from roads. Consequently, we found no reasonable explanation for the influence of road density in the conservative model and conclude that it was a spurious result of small sample size.

### 4.4. Structure of Coati Distribution at its Range Margin

Species distribution models represent the range of conditions under which the species may occur and they provide no inherent information about how the species might respond to different habitats or environmental conditions in terms of abundance, reproduction, survival, or other ecological traits. Consequently, they are almost always an over-prediction of the actual area occupied by the species at any given time, which may be due to factors such as interspecific interactions, microhabitat requirements, or metapopulation structure that limit occurrence. This is particularly true at range margins, where distribution patterns are spatially complex and temporally variable [51]. Rarely is there a simple range margin in which there is an abrupt transition between where a species occurs and does not occur. Rather, the spatial structure of a range margin may consists of several zones from interior of range to exterior of range, including: (1) zone where species is continuously distributed; (2) zone where species persists as disjunct populations in patches of favorable environment; (3) zone where temporary disjunct populations may form during periods of favorable conditions or due to recurrent immigration; and (4) zone where individuals may disperse but do not survive [52,53]. 

Given the topographic and environmental variability of the study area, it seems likely that coatis would exist in this region as metapopulations and likely conform to the four spatio-temporal range margin zones. Consequently, it would be desirable to know which habitats serve as potential source populations where birth rates exceed death rates. The white-nosed coati has an unusual social system. Females and juveniles form year-round social bands, although reproductive females are solitary for a ca two month period surrounding parturition which occurs in early July [54]. In contrast, adult males are solitary most of the year, although home ranges usually overlap that of their natal band [47,55]. However, during the mating season (mid-March to mid-April in Arizona) adult males may disperse to and temporarily join unrelated bands in order to avoid inbreeding [55]. However, there are no published data that indicate the maximum dispersal capability of adult males during this time. Further, evidence from Arizona suggests that yearlings and adults of both sexes may disperse away from their natal groups and ranges in this region [49]. Some studies have suggested that extralimital records represent wandering males [21]. Other studies have suggested that bands may be semi-nomadic, based on observations of temporally intermittent occurrence of coati bands at specific locations and observations of coati bands shifting core use areas [19]. In contrast, other studies have found that band home ranges in Arizona only slightly shifted from year to year [47].

To evaluate possible breeding distribution of the coati in the study area, we mapped locations of observations of coatis that included juveniles, pregnant females, or groups of three or more individuals, and made the assumption that such observations represented bands. Bands were found primarily associated with isolated mountain ranges in southeastern Arizona and southwestern New Mexico, in a belt around the mid-elevations of the southern and western edge of the Mogollon Highlands, and scattered locations on the Rio Grande and Organ Mountains in south-central New Mexico (Figure 4). The majority (44%; Table 6) of bands were in Madrean land-cover types, which appeared to be the preferred habitat for coatis in this region. However, bands also were regularly associated with various Chihuahuan land-cover types, especially in New Mexico (32%; Table 6). This might reflect the fact that Chihuahuan semi-desert grassland typically borders Madrean land-covers at their lower elevation border in this region. However, it also might indicate that coatis are more capable of living and reproducing in Chihuahuan habitats than previously recognized. We also evaluated the season for seeming extralimital records of coatis from the Central Highlands and Colorado Plateau in Arizona (*i.e.*, Apache, Coconino, and Yavapai counties). Of nine records with dates, all were of single animals including two in June, one in July, three in September, one in November, and two in January (one known to be male). Given the dates, none of these records are likely males dispersing during the mating season; and the six fall and winter records are almost certainly not solitary reproductive females. The provenance of these occurrences remains unknown.

**Table 6:** Percent of observations of bands of white-nosed coati (*Nasua narica*) in various land-cover types in Arizona and New Mexico.

Land-cover	Arizona (N = 13)	New Mexico (N = 90)	Total (N = 103)
Madrean Pinyon-Juniper Woodland	15.4	28.9	27.2
Apacherian-Chihuahuan Piedmont Semi-Desert Grassland and Steppe	0.0	20.0	17.5
Madrean Encinal	30.8	11.1	13.6
Mogollon Chaparral	15.4	6.7	7.8
other	0.0	8.9	7.8
Apacherian-Chihuahuan Mesquite Upland Scrub	7.7	6.7	6.8
Rocky Mountain Ponderosa Pine Woodland	7.7	6.7	6.8
Chihuahuan Mixed Salt Desert Scrub	7.7	4.4	4.9
Madrean Pine-Oak Forest and Woodland	7.7	2.2	2.9
Chihuahuan Creosotebush, Mixed Desert and Thorn Scrub	0.0	2.2	1.9
Chihuahuan Stabilized Coppice Dune and Sand Flat Scrub	0.0	1.1	1.0
Colorado Plateau Pinyon-Juniper Woodland	0.0	1.1	1.0
Sonoran Paloverde-Mixed Cacti Desert Scrub	7.7	0.0	1.0

We propose that coatis in the American Southwest serve as an ideal model for studying structure and dynamics of species range margins. Available evidence suggests that coati populations in well-developed Madrean habitats, such as the Huachuca Mountains, form bands that are relatively spatio-temporally stable such that presence is predictable [47]. Because of the disjunct nature of quality Madrean habitat on isolated mountains, it seems likely that these form a metapopulation (*i.e.*, range margin zone 2). However, in less ideal habitats in this region, evidence suggests that the presence of potential bands is less predictable. For instance, groups and juveniles have been reported on several occasions from the Rio Grande and Organ Mountains in New Mexico, but these occurrences appear to be intermittent and hence occurrence of coatis at any given time or place is not predictable (*i.e.*, range margin zone 3). Finally, records of individual coatis of mostly unknown gender occasionally occur in areas extralimital to the known breeding range. These records are difficult to account for based on known demographic characteristics of coatis. For instance, predation can account for high mortality rates in coatis, especially for solitary animals [56]. Demographic studies have not documented coatis living truly independent existences that are significantly spatially removed from bands. Consequently, it remains unknown if the apparent extralimital records represent dispersing individuals (*i.e.*, range margin zone 4), or perhaps represent rare and intermittent occurrence of bands in margin habitat (range margin zone 3). Field studies of these occurrences will be required to understand the situation.

## 5. Conclusions

**Figure 5:**
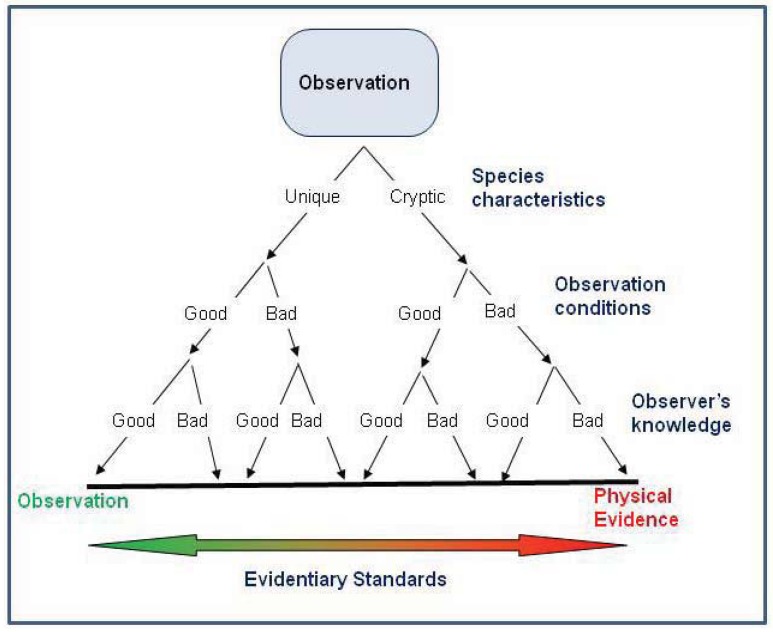
Scheme of evidentiary standards for occurrence records based on the species characteristics, observation conditions, and observer’s knowledge. The highest evidentiary standards (*i.e.*, requiring physical evidence) are necessary when the species poses identification problems or when observation conditions or the observer’s knowledge are poor. In contrast, anecdotal evidence might be acceptable for interpreting distribution if the species has readily observable diagnostic features, especially if observation conditions allow evaluation of the diagnostic features, or the observation is made by a taxon expert.

This study is the first to evaluate the impact of reliability of occurrence records on niche-based species distribution models. We found that for the white-nosed coati, inclusion of anecdotal records provided similar results compared to those based only on verified records. Thus, field observations may provide an important source of data for understanding the distribution of many rare species where there is a paucity of physical evidence. This is important because anecdotal data can provide some benefits over physical evidence such as being relatively inexpensive, abundant (possibly providing better geographic coverage), and derived from multiple sources (possibly negating some sampling biases). In contrast to McKelvey *et al.* [1] who suggested that higher evidentiary standards should used for determining the distribution and status of rare or elusive species, we believe that higher evidentiary standards are required for species that pose identification problems, and where observation conditions or observer knowledge are poor (Figure 5). However, we strongly caution that our results may not be applicable to all situations. We recommend that anecdotal occurrence records only be used according to the following criteria: (1) Maximum entropy methods should be used to infer distributions based on anecdotal data because the algorithms assign low probabilities to unusual occurrences. (2) Occurrence records should be evaluated for their reliability with only the most reliable used for interpreting distribution. (3) Anecdotal records should be used to supplement (not in lieu of) physical evidence. (4) Species must exhibit readily observable diagnostic features; cryptic species require either physical evidence or observation and verification by a taxon expert. Lastly, we urge for additional research on the influence of the reliability of occurrence records on species distribution models, especially using simulation data and making comparisons among species that vary in ease of identification and comparisons among different demographic groups of observers (e.g., experts *versus* naïve).
